# Learning to Wait and Be Altruistic: Testing A Conversational Training in Economic Education for Primary School Children

**DOI:** 10.5964/ejop.2453

**Published:** 2021-11-30

**Authors:** Elisabetta Lombardi, Annalisa Valle, Teresa Rinaldi, Davide Massaro, Antonella Marchetti

**Affiliations:** 1 Theory of Mind Research Unit, Department of Psychology, Università Cattolica del Sacro Cuore, Milan, Italy; University of Wroclaw, Wroclaw, Poland

**Keywords:** decision making, training, altruism, intertemporal choice, investment, fairness, school-age children

## Abstract

Individual economic competence is important but increasingly challenging to manage due to the growing complexity of the nature of economic decisions people must make and the substantial impacts of some of these decisions on their lives. Decision-making ability develops from childhood and is closely related to specific economic components and prosocial behaviour such as fairness, altruism, and delay of gratification. However, while there are financial-education programs for children and young people focusing on financial products, few studies have examined training for the psychological abilities underlying economic decision-making. To promote those psychological skills that contribute to a more socially effective decision-making, we designed and tested a conversational-based training program for primary school children using reflective thinking. A total of 110 (male = 47, female = 63) children aged 8 to 10 years (Mean age = 9.71 years) from two schools in Northern Italy participated in the study with 55 children in a training group and 55 in a control group. All participated in pre-tests measuring their socio-economic background and economics-related skills and abilities. The training group were told stories relaying values of fairness, altruism, and delayed gratification. Both groups participated in task-based post-tests relating to fairness, altruism, and delayed gratification. Results revealed that children in the training group showed significant improvement at the post-test in altruistic and investment behaviour, showing the training efficacy, suggesting that similar programs could be implemented in primary schools as foundational teaching of economics and fiscal responsibility.

Economic education has become an increasingly important issue in the last decade, due to the numerous changes in the economic and social context. Literature has aimed at investigating economic and financial phenomena, particularly financial literacy (Lusardi & Mitchell, 2014), evidencing that a lack of economic-financial knowledge is disadvantageous to people lives (Bucher‐Koenen et al., 2017). Lower levels of such knowledge, as in the case of women, have an impact on the active participation within the economy, also within the household (Hung et al., 2012), and makes people vulnerable. On the contrary, high levels of financial literacy result in positive economic outcomes, that is, planning for retirement, paying bills on time, budgeting, saving, and setting financial goals (Grohmann et al., 2015), and positively correlate with day-to-day financial management skills, the participation in financial markets and investments and the capacity to undertake a retirement planning. These evidences highlight the need for providing children and young people with effective financial education programs since an early age to prepare them for understanding and experiencing the economic and financial occurrences (Aprea, 2015; Berti et al., 2017; Lombardi & Ajello, 2017).

The Organisation for Economic Co-operation and Development (OECD; OECD, 2014) defines financial literacy combining three aspects: knowledge of financial concepts; financial capacity (the ability to apply this knowledge in real life); and financial inclusion (describing the opportunities and motivations for inclusion in various financial scenarios). The second aspect directly connects to decision-making—a psychological process relevant to improve good financial literacy. In fact, both the first definition of financial education (OECD, 2005) and the most recent literature identifying the key features of financial education programs (Amagir et al., 2018) focusing on the importance of being able to make appropriate economic and financial choices to achieve positive economic behaviours. Decision-making is a complex process, involving a number of psychological constructs, such as fairness, altruism, and the ability to delay a gratification; as for childhood, literature focuses on developing and educating decision-making skills in order to better manage goods, money and to become able to understand economic world (Castelli et al., 2014; Castelli et al., 2017; Lombardi et al., 2017; Marchetti et al., 2016).

Fairness can be defined through the inequity aversion concept (Fehr & Schmidt, 1999), that is, people’s tendency to resist inequitable outcomes. In economic transactions, fairness can lead people to give up possible profits in order to re-establish equity. This is considered a strategic approach to economic decision-making, because increases over time the chance of reciprocity: an individual can currently give up part of her/his assets to another knowing that in the future she/he will be treated fairly, thus gaining an advantage. The main task evaluating fairness is the *Ultimatum Game* (UG), an economic interactive game involving one Proposer and one Receiver that have to share an amount of money. Fair Receivers accept fair offers, in which the amount of money is similar for the two players, and refuse unfair offers, in which one of the players receives significantly more money than the other. Concerning childhood, around 3–4 years of age, children show aversion to disadvantageous inequity by rejecting offers that provide for a lower good for oneself and a higher good for the other; around 8 years of age, they show aversion also to advantageous inequity, rejecting offers that provide for a higher good for oneself and a lower good for the other (Smith et al., 2013). Thus, the baseline for fairness shifts from an egoistic/egocentric perspective, oriented to maximize profit without considering others’ perspectives, to an equal/multicentric perspective, which allows children to play considering the partner perspective on the fairness norm (Castelli et al., 2017).

Altruism is a predisposition of human beings to help others achieve their goals and to share valuable goods, services and information, with the long-term aim to improve the society well-being and consequently also one's own (Warneken & Tomasello, 2009). Children learn to act altruistic behaviours on the basis of their own culture’ social norms, expecting of being reciprocated and thinking to their social reputation. Altruism is studied by the *Dictator Game* (DG), where the Proposer decides how much to offer to the Receiver, who is obliged to accept. Children start helping others and share with others already during the second/third year of life (Warneken & Tomasello, 2009, 2013), then propensity to altruism becomes stable at early school-age (Benenson et al., 2007).

Furthermore, people are often called to make decisions between choices that have an immediate benefit and choices that have a greater benefit in the future. This decision is named “intertemporal choice” and regards the behaviour to act when choices in the present influence future availabilities, as in the case of saving, investment, education, health care. Investigated through the delay of gratification paradigm (Marchetti et al., 2014), the ability to wait for a higher award affects developmental psychology, because predicts school context adaptation, attainment of academic achievement, high salaries and good job positions in adult life (Casey et al., 2011). This ability surfaces at preschool age (a turning point is around four years) and continues to develop until 8–10 years of age, when children can inhibit an immediate impulse in order to obtain future gains (Lombardi et al., 2017).

## Why a Conversational Training for Decision-Making Components?

Analysing financial-literacy education programs, Amagir and colleagues (2018) show that these programs usually teach basic concepts and/or content of the economic and financial world. Authors argue that an educational approach based exclusively on knowledge has limited effectiveness (Perry & Morris, 2005): in order to obtain a significant improvement is important to consider the financial capability. Hence, several existing programs focus training on some of personal aspects involved in economic and financial decision-making (i.e., self-confidence, perseverance, and “economic thinking,” but also mathematic competency), transferable skills, willingness to invest in oneself to achieve economic improvements, and problem-solving skills. To become a good decision-maker (making effective decisions on a personal level that are socially acceptable from an interpersonal point of view) is important making adaptive long-terms decisions, depending on a person’s planning skills, ability to wait, and capacity to delay a gratification, all abilities studied in psychology as processes underlying the development of individuals’ social skills. Moreover, a large part of daily decisions are the basis of the prosocial behaviour—costly to the individual and benefits others at the individual or group level (Yamagishi et al., 2012); examples include altruism, charitable donations, and helping behaviours. Böckler and colleagues (2018) identify three factors constituent prosocial behaviour that can be trained: *altruistic motivated prosocial behaviours* (demonstrating individual desire to enhance other’s well-being even at a cost to oneself and evaluated through, for example, the donation task or the DG); *norm motivated prosocial behaviours* (the tendency to enforce social norms using costly punishment) evaluated through second and third-party punishment tasks (a variation of the UG); *self-reported motivated prosocial behaviours* (perceiving oneself as moral and helpful) evaluated through self-reported scales. More specifically, the trainings concerning prosocial behaviours focus on: individual affective components, that is, compassion, gratitude, prosocial motivation; socio-cognitive skills, that is, perspective-taking ability; mindfulness, that is, compassion-based contemplative practices. These trainings may involve adults (parents or teachers) to train or to teach specific strategies to use with children or adolescents (e.g., Šramová, 2004; Valle et al., 2016) or may be applied directly children and adolescents. With regard to the latter, Heck and colleagues (2018) proposed a training for primary school children focusing on the construct of fairness, demonstrating that training children in perspective-taking, influences their decisions in economic games.

In light of these considerations, we involve primary school children in a conversation-based training to enhance prosocial behaviour and competencies by developing perspective-taking abilities. This conversational training applies methods used by financial education programs, such as group discussion and guided readings (Amagir et al., 2018), and focuses on metacognitive ability to think about self and perspective-taking ability (Böckler et al., 2018). Our training use conversations as a means of co-constructing knowledge (Siegal, 1999): children are guided to discuss each other’s, with the aim of discovering and accepting multiple perspectives, in order to compare different points of view and promote reflection on experiences (Durlak et al., 2011). In this way, this training supports the application of the decision-making and its components, that is, altruism, fairness, and intertemporal choice, in children’s daily life.

The aim of this study is to evaluate the effectiveness of the conversation-based training in fairness, altruism, and delay of gratification ability on economic decisions in children from 8 to 10 years old. We hypothesize that reflections facilitated by a conversational methodology on the issues above-mentioned will lead children to change their behaviours in decision-making from pre- to post-test, compared to children in the control group (CG). We expected that children evaluated at the end of the training would show more inequity aversion in the fairness test, would become more altruistic and better able to wait for a greater good than in the pre-test evaluation with respect to children of the CG.

## Method

### Participants

Initially 121 children were recruited for this study belonging to six classes (from 3rd to 5th primary school classes) from two schools in Northern Italy, near Milan, who took part in this study. Children who did not complete all the measures or children did not speak or understand Italian were removed from the main dataset. Six children assigned to the training group (TG) and three children assigned to the control group (CG) didn’t complete pre- or post-test sessions and two children, assigned to CG, had moved to Italy for no more than 3 months and did not understand or speak Italian. The total of participants was 110 (male = 47, female = 63) aged between 8 to 10 years (*M*_age_ = 116.51 months, *SD* = 10.49 months). Two classes for each age range participated and for every range one class was randomly assigned to the CG (*N* = 55, *M*_age_ = 118.15 months, *SD* = 10.31, male = 26, female = 29) and one to the TG (*N* = 55, *M*_age_ = 114.91, *SD* = 9.80, male = 21, female = 34). The TG participated in the training program, while a CG followed only the regular school program of citizenship education. Children was made up of typically developing who were fluent in Italian and had not difficulties in taking part (and learn from) the activities of our training program. Parental informed consent was obtained from each participant. The research was conducted according to APA ethical standards and was approved by the local ethics committee.

### Procedures

The study was organized into three steps:

**Step 1 (Pretest):** All children were tested firstly through a collective session and secondly through an individual one. The collective session, lasting about 50 minutes, included a guided-by-the-experimenter protocol to assess socio-economic families’ level, linguistic and mathematical abilities of the children. The individual session tasks were randomized and evaluate children's inhibitory control, sensibility of fairness, altruism and the delay of gratification. During the two individual sessions, lasting about 25 minutes, children could play with and had the chance to win football players or puppies trading cards used as traded goods for the proposed games. Before starting each task, children were asked about their trading cards preferences. Each task was presented randomly.

**Step 2 (Training):** Only those children in the TG took part in the training sessions, which started one week after the end of the pre-test phase. Children in the CG only attended civics education classes, established in their state curricula. Both training and CG followed the school curriculum based on the Italian National Guidelines for the pre-primary school and the first cycle of school education curriculum (Ministero dell’Istruzione, dell’Università e della Ricerca [MIUR], 2012). It indicates that the general objective of the educational process in the school system is the achievement of some key competences for lifelong learning recommended by the European Parliament and the Council such as the sense of initiative and entrepreneurship, strictly linked with economic and financial education. According to these guidelines, every teacher individually and in a personal way shows the principles of the economic and financial education, explaining, for example, the economic trend of industry sector (Morselli & Ajello, 2016).

**Step 3 (Post-test):** All children took part in this session one week after training sessions end. They only attended the individual session in which they were re-tested about fairness, altruism and delay of gratification. Tasks were run in random order during one individual session lasting a maximum of 25 minutes. The post-test session ended at the end of the school year, after 4 months from the pre-test session.

Both pre-test and post-test individual sessions were conducted in a quiet room different from children's classes. The training sessions were conducted in the classroom. The three steps of researcher were conducted by independent researchers. As shown in Table 1, we organized the variables in “control variables,” potentially confounding variables that are known to be related to fairness, altruism and delay of gratification and “decision making variables,” focus of the intervention. Decision making tasks were played for real, giving a final amount of trading cards.

**Table 1:** Target Dimensions and Tasks for the Pre-Test and Post-Test Administrations

Type of variable/Dimension	Task	Pre-test	Post-test
Control variables			
Socio-economic background	Family Affluence Scale (FAS, Currie et al., 2008)	X	
Verbal ability	Primary Mental Ability (PMA, Rubini & Rossi, 1982; Thurstone, & Thurstone, 1982)	X	
Mathematical ability	AC-MT 6-11 (Cornoldi et al., 2012)	X	
Inhibitory control	Fruit Stroop Task (Archibald & Kerns, 1999)	X	
Decision making variables			
Fairness	Ultimatum Game (UG)	X	X
Altruism	Dictator Game (DG)	X	X
	Donation Task (DT)	X	X
Delay of gratification	Intertemporal Choice Task	X	X
	Investment Task	X	X

### Decision-Making Variables

#### Fairness

A modified version of the UG (Güth et al., 1982) was used to assess fairness. Children played a game in which they could be shared with another child represented by a drawing image up to 10 trading cards. Playing the role of Receiver, the child could decide whether to accept or refuse the proposed division. The children played three rounds as Receiver categorized as follows: *unfair* (8–2: eight trading cards for the Proposer and two trading cards for the Receiver); *hyperfair* (2–8: two trading cards for the Proposer and eight trading cards for the Receiver); and *fair* (5–5: equal division). All rounds were presented randomly. The children scored 1 when the offer was accepted and 0 when refused. A total of 3 independent scores were hence obtained, one for each type of offer.

#### Altruism

The DG (Kahneman et al., 1986) and the Donation Task (DT; Angerer et al., 2015) were used to assess altruism. In the DG, the child (playing as Proposer/Dictator) decided how to distribute 10 trading cards, between him and a passive player, that did not have the option to decline the offer. Also, in this case, the other child (the Receiver) was presented as a drawing image and the Dictator has chosen between two different typologies of trading cards. The children played only one round, in which the offered amount was scored.

Based on the donation experiment run by Angerer and colleagues (2015), we used the DT, that is, a DG-like experiment on donations to a charity. The experimenter first asked the child if he/she could see a box placed on the other side of the room. Once the child replied “Yes,” the experimenter began to explain to him/her that the box contained all the trading cards donated by the children participating in the project to some children whose families didn’t have money to buy them. Then the experimenter told the child he/she would have had 10 trading cards and he/she could decide how many of them donate and how many taking home. The child was informed that he/she could donate from 0 to 10 trading cards, inserting the donated cards in the box. Cards he/she would take home had to be put in a white envelope, without being observed by anyone. After a couple of control questions on the understanding of the right donated and taken-home trading cards’ allocation, the experimenter accompanied the child in front of the box and gave him/her all the time waiting for him/her in another part of the room. Scores could vary from 0 to 10, depending on the number of trading cards donated.

#### Delay of Gratification

The Intertemporal Choice Task (ICT-version of Marchetti et al., 2014) and the Investment Task (IT; Angerer et al., 2015) were used to assess the delay of gratification (Mischel et al., 1972). In the ICT, the experimenter asks the children to decide whether they want to delay gratification in hopes of gaining larger future reward. Children were first told the following sentence: “You know, sometimes you can choose between receiving a small gift right away or a bigger one later” and then they had to answer the following question: “Do you prefer having a pack of trading cards now or wait four weeks, the day XX (showing the right day on a calendar) to have two trading cards’ packs?.” In case the child chose to take one pack of trading cards immediately, he was asked how long he would be willing to wait to get two packs. The experimenter took to school in the right day after four weeks trading cards children won. The child scored 0 if could not wait four weeks and 1 if waited.

The IT (Angerer et al., 2015) assess the investment propensity as a part of the delay of gratification paradigm. Compared to the former task, the IT requires to apply a more strategic thinking in the decision to delay an immediate gratification in favour of a greater future reward, because the child has to decide how many trading cards to take home immediately and how many to invest. In this case, the child has to manage the pursuit of two objectives, one immediate and one long-term, assessing whether and how much more important for her/him the immediate reward or the greater future reward is. In fact, in this task children were endowed with 10 trading cards and they were told they had to choose how many trading cards they could take home immediately and how many they want to put inside of “four weeks” box. Every card inserted in the box would have been doubled if children would have waited for four weeks (children had been shown the exact day on a calendar). To understand children's rule comprehension, they were asked to repeat it with some control question. Once the children real comprehension was verified, they were told to make their choice. The score was the invested trading cards number (range 0–10). The experimenter took to school in the right day after four weeks trading cards children invested.

### Training

A new conversational training focused on fairness, altruism, and delay of gratification was created in order to train these skills. The conversational approach (Siegal, 1999) assumes that child is involved in conversational interactions, typical of social life, early in development. The conversational activity, in particular during the school-age period, allows transforming the implicit knowledge into explicit knowledge, discussing them with others.

The training was designed to have three one-hour sessions each, conducted in class by a researcher over a period of about two weeks of school time. For each topic (i.e., fairness, altruism, and delay of gratification ability), two stories have been invented or created based on children's (Varela, 2014) or on scientific literature (Larsen et al., 2017), with the aim of stimulating group reflection and understanding of one's own and other points of view. According to literature about the training programs (Bianco et al., 2019; Bianco et al., 2021), each story was followed by four multiple-choice questions create with the purpose of verifying child's actual understanding of the content, his/her ability to put themselves in the shoes of the story characters (perspective-taking) and to stimulate the subsequent discussion.

## Results

Performance on the ICT as well as on the UG was evaluated through non-parametric statistics (binomial analysis and Mann-Whitney *U* test). We conducted some preliminary analyses to verify the homogeneity of the groups for the considered variables at the pre-test session. We controlled gender differences and no significant results emerged. To assess differences in the pre-test rate of acceptances of hyperfair, fair and unfair proposals and of ICT’s success the Mann-Whitney *U* test (Bonferroni corrected for multiple comparisons) by paired-group showed no significant differences between the two groups (*p* > .05). For the other variables, we conduct the *t*-test for independent samples and it didn’t show any statistically significant differences between children assigned to the TG and children assigned to the CG (*p* > .05), with exception of the verbal abilities, *t*(108) = 2,376, *p* = .019. For this significant difference in subsequent analyses, we controlled verbal abilities scores.

**Figure 1:**
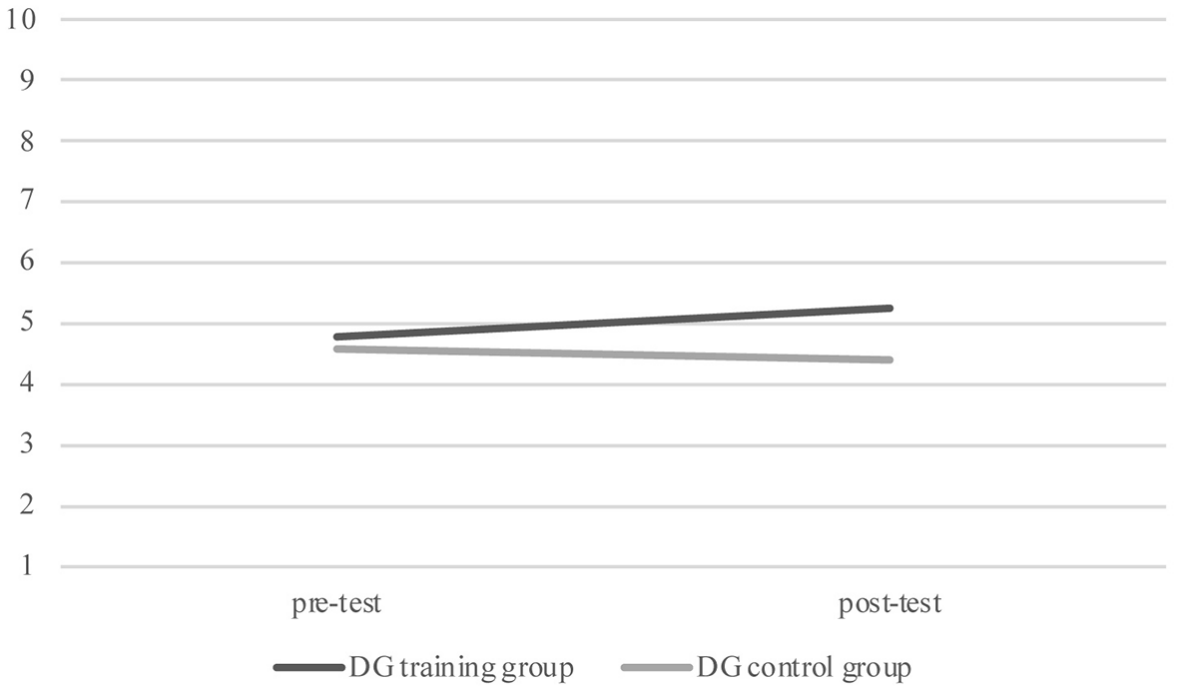
Dictator Game Proposals for Training Group and Control Group at Pre-Test and Post-Test

**Figure 2:**
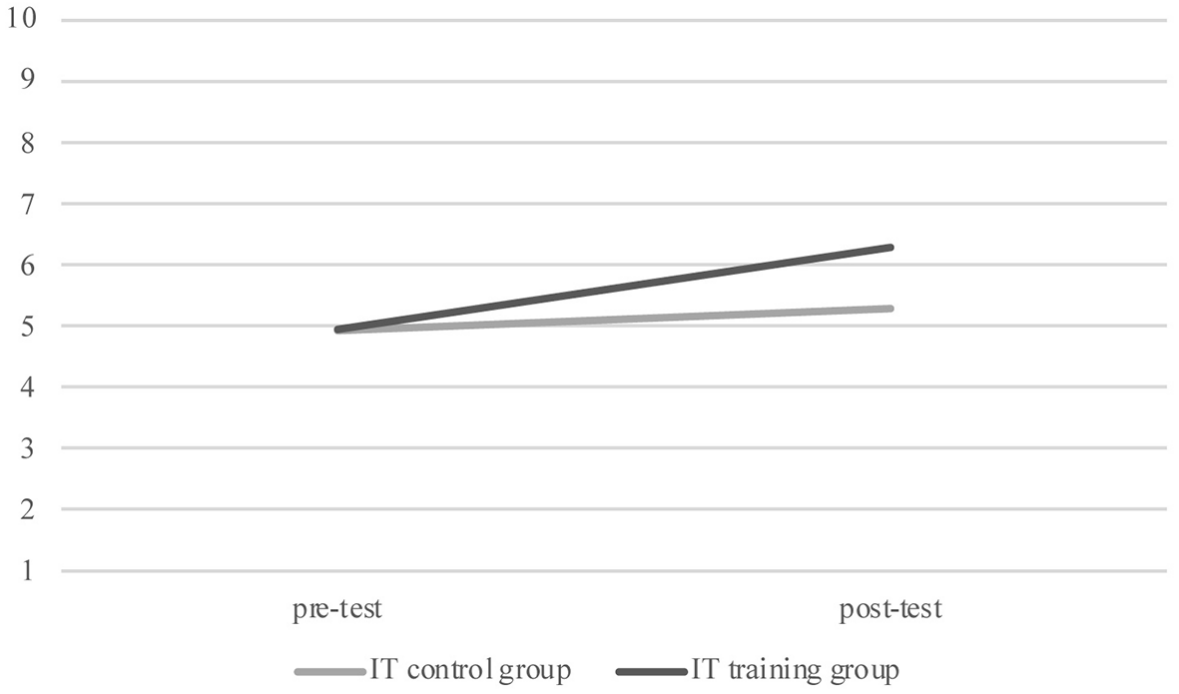
Investment Task Performance for Training Group and Control Group at Pre-Test and Post-Test

Subsequently, in order to analyse the effect of training, we performed a GLM for repeated measures for each decision-making continuous variable explored, that is, DG, DT, IT with time (pre-test and post-test) as the within-subjects factor and groups (training and control) as the between-subjects factor, and verbal ability as the covariate. Pairwise comparisons revealed that, as shown in Figure 1, for the DG, children in the TG showed significantly higher post-test offers compared to the post-test offers in the CG, *F*(1, 108) = 5.431, *p* = .022, η^2^ = .071, θ = 0.700. Furthermore, for the IT children in the TG showed a significantly higher post-test investment compared children in the CG, *F*(1, 108) = 4.270, *p* = .041, η^2^ = .038, θ = 0.535, showing the efficacy of the training program (see Figure 2). However, for the DT, GLM for repeated measures does not show significant effect of training, *F*(1, 108) = 0.143, *p* = .706, η^2^ = .006, θ = 0.130. In order to evaluate the effect of training for the dichotomous variables, that is, the UG—fair, unfair and hyperfair proposals—and ICT, we used the McNemar’s statistic in the two groups. This test was significant for both CG and TG for the ICT (TG, *N* = 55, χ^2^ = 10.9, *p* < .001; CG, *N* = 55, χ^2^ = 10.9, *p* < .001), showing an effect of the time and it was no significant in the two groups for UG fair proposal (TG, *N* = 55, χ^2^ = 0.40, *p* = .527; CG, *N* = 55, χ^2^ = 0.50, *p* = .480), UG unfair proposal (TG, *N* = 55, χ^2^ = 0.258, *p* = .108; CG, *N* = 55, χ^2^ = 0.07, *p* = .796) and UG hyperfair proposal (TG, *N* = 55, χ^2^ = 1.0, *p* = .317; CG, *N* = 55, χ^2^ = 0.82, *p* = .366). These results show that the training had no efficacy in the performance of these tasks.

## Discussion

In this study, we tested the efficacy of a conversational training about fairness, altruism, and the ability to delay gratification in children aged from 8 to 10 years. Results evidence that the training increases altruistic behaviour and the ability to delay gratification, whereas does not impact the fairness behaviour.

Regarding the altruism increase, the literature suggests that the propensity for altruism is already seen in early childhood (Warneken & Tomasello, 2009) and stabilizes in early school-age (Benenson et al., 2007). Nonetheless we find that the training modifies altruistic behaviour in the late school-age: children who participated in the conversational training increase the number of the trading cards shared in the DG, but they didn’t increase the number of the trading cards donated in the DT. The latter explicitly evokes the construct of charity (a concept similar to that of a donation considered here) consisting of resources allocation to a recipient identified by need, not by personal characteristics (Niemi & Young, 2017). The DG requires children to play with another hypothetical—but well defined—child, because of a schoolmate depicted in a drawing, whereas the DT asks to share some trading cards with an unfamiliar child. It is possible that children trained in the perspective-taking with their classmates become more able to assume the perspective of a specific child similar to them, then they based the choice of the number of trading cards to share on the assumption of a hypothetical relationship with her/him. In the DT, charitable behaviour is based on the identification of a need, without implying or hypothesizing a direct relationship with the other; consequently, in this case the ability to take others’ point of view may be less involved.

Regarding the ability to delay gratification, children of the TG increase the number of trading cards invested in the IT, compared to the CG, but we do not find differences in the ICT. In the ability to delay a gratification are involved self-control (Kidd et al., 2013), used to inhibit the desire to obtain the gain immediately, anticipation, the capacity to anticipate the hedonic consequences related to the good in the future, and representation, the tendency to evoke specific interpretative frames about the salience of the delayed reward (Berns et al., 2017). We assumed that the application of these capacities during the training helped children to become more strategic in an IT, a complex situation that involves the ability to anticipate and represents both the immediate and the future gain and that requires to find an equilibrium between them (both ensured, the decision is about the amount of the rewards). Conversely, the intertemporal choice is less complex and less strategic because imply an “all or nothing” decision (a reward immediately or a reward in the future), then it is possible that children continue to apply their usual behaviour without benefiting from more complex reasoning.

Regarding fairness, we had assumed that after participating in a training focused on the fairness norm, children showed more inequity aversion that in the pre-test phase, by the increase of the rejections of unfair and hyperfair offers. Instead, results suggest that the training did not have an effect on the inequity aversion, in both directions. To understand this result it is useful refer to the overlapping of the concepts of fairness and inequity aversion: indeed, the fact that to train fairness does not impact on inequity related behaviour may mean that in this age groups, social norm of fairness is something different from its behavioural operationalization in inequity aversion. This hypothesis is in line with a recent work of Engelmann and Tomasello (2019), affirming that children decide about the resources’ allocation on the basis of the social meaning attributed to this distribution and specifically on the basis of the desire that people are equally respected. In this perspective, children’ decisions are not moved by an abstract norm of fairness (object of the present training), rather by the application of this norm involving an interpersonally based reasoning on the mutual respect, the merit (in the case of collaboration) and the resource’s need. We can assume that to obtain a change in the economic behaviour it might be useful to work on these social aspects, rather than on the norm itself, as proposed during the training.

Finally, results showed that using guided conversations and training children to focus themselves on the reflective thinking about norms, values and possible different perspectives, altruism and investment decision-making behaviour are modified. Reflective thinking can help to monitor and display the solution/decision process, through the problem-solving with logical reasoning, in order to analyze and think about the options, choosing the most useful alternative. Decision-making requires to reflect knowingly on their own mental structures and procedures, emerging as a solution to interpret, delay and understand the issues of thinking in prediction and decision-making for the future (Rasyid et al., 2018). We think that reflective thinking supports reflections and discussions and helps children to develop higher-order cognitive skills through the link of the new to their previous knowledge, the implementation of specific strategies for new tasks and the aware understanding of their own thinking processes and decision strategies. Many studies showed how learning occurs through social and communicative processes, as forms of "dialogic" interaction, such as classroom discourse (Mercer & Littleton, 2007). In the training, each child discussing with other participants recognizes the diversity of voices, values, beliefs and perspectives and the meaning emerges from the tension between the perspectives in that "dialogic space" which develops through the social construction of meaning (Lombardi et al., 2018; Perret-Clermont et al., 1991). Training helps children to reflect on their own thoughts and decision-making. Participating in shared reasoning and thoughts, and critically considering other points of view were useful to learn and generalize new forms of thinking. At the end of this training, new knowledge in children derived not only from materials prepared by the researcher, used just as a stimulus to start the discussion, but also from listening to mutual comparison, in a more active and interesting way. Furthermore, children learn something about the topic and something about aspects of this topic related to their social world and, putting themselves in the story protagonists’ shoes, they may change their decisions. This is particularly important due to the relevant influence on children’s development of symbolic representation of other’s mind (Giovanelli et al., 2020). Children rely on previous knowledge and work to actively welcome new information to make sense of the story situation; they move from considering the concrete, action-oriented, context-specific details of the stories to building an understanding of the wider and longer-term emotional implications for their own situation (Immordino-Yang, 2015). The training may also have stimulated cognitive processes underlying thoughts and behaviours regulation in children, such as cognitive flexibility, refers to our ability to switch between different mental sets, tasks, or strategies (Diamond, 2013). The TG children refocus attention to relevant theme of the training session and simultaneously consider conflicting representations of information in order to modify one's thinking in response to changes in their own internal or external environment and in relation to their decisional process.

### Limits, Strenghts and Conclusions

About the limits of this study, in the future it will be important to let children play as proponents of the UG: in fact, literature evidences that school-age children evaluate differently the fairness of the offers when they play as Proposer or Receiver (Castelli et al., 2014). It might be interesting to check whether playing as a Receiver can bring changes that are not appreciable when the children play as Proponents. Moreover, we did not evaluate the trust in the experimenter role: an experimenter tested all children in the pre-test and post-test phases, and she came back to deliver the gained trading cards during the games. It is possible that to verify the experimenter’s reliability in the first phase has led the children to trust that person even in the second phase, influencing in some way decisions in the post-test (about the importance of the reliability of the experimenter see Kidd et al., 2013). From the methodological point of view, another limit concerns the difficulty of discriminating the effect of learning in the post-test session, although the TG is significantly improved compared to the CG. In future studies, will be useful consider the transfer effect of our training in order to test its efficacy in producing improvements on practiced but also on transfer tasks. Moreover, the two groups followed normal school programs, future research should use a control training with the same structure as the experimental one, but with neutral contents.

A strength of the training concerns the applicability in the educational context in order to improve both specific and broad psychological dimensions. In fact, results showed that a training applying school methods, familiar for teachers and pupils, have an impact on very specific dimensions such altruism and delay of gratification, but also may promote more general psychological abilities, for example reflective thinking as discussed above.

In light of our results, we think that the application of this training at school might be useful for teachers and children. The training’s structure, based on narratives’ stimuli and guided discussion, is near to the teaching methods usually used at school, they might be easily accepted and applied in a classroom. Moreover, this training does not directly refer to the subject of economics, which is generally not included in primary school curricula, but its application provides foundational learning related to economic topics for this age group.

## Supplementary Materials

For this article Appendices 1 and 2 are available via PsychArhives (for access see Index of Supplementary Materials below):

- Appendix 1: Decision making training.
- Appendix 2: Descriptive statistics, binomial analysis and correlations.

## Author Note

The first two authors are the main contributors to the writing of this paper.
